# Stage-independent, single lead EEG sleep spindle detection using the continuous wavelet transform and local weighted smoothing

**DOI:** 10.3389/fnhum.2015.00181

**Published:** 2015-04-08

**Authors:** Athanasios Tsanas, Gari D. Clifford

**Affiliations:** ^1^Department of Engineering Science, Institute of Biomedical Engineering, University of OxfordOxford, UK; ^2^Wolfson Centre for Mathematical Biology, Mathematical Institute, University of OxfordOxford, UK; ^3^Nuffield Department of Medicine, Sleep and Circadian Neuroscience Institute, University of OxfordUK; ^4^Department of Biomedical Informatics, Emory UniversityAtlanta, GA, USA; ^5^Department of Biomedical Engineering, Georgia Institute of TechnologyAtlanta, GA, USA

**Keywords:** decision support tool, hypnogram, signal processing algorithms, sleep spindle, sleep structure assessment

## Abstract

Sleep spindles are critical in characterizing sleep and have been associated with cognitive function and pathophysiological assessment. Typically, their detection relies on the subjective and time-consuming visual examination of electroencephalogram (EEG) signal(s) by experts, and has led to large inter-rater variability as a result of poor definition of sleep spindle characteristics. Hitherto, many algorithmic spindle detectors inherently make signal stationarity assumptions (e.g., Fourier transform-based approaches) which are inappropriate for EEG signals, and frequently rely on additional information which may not be readily available in many practical settings (e.g., more than one EEG channels, or prior hypnogram assessment). This study proposes a novel signal processing methodology relying solely on a single EEG channel, and provides objective, accurate means toward probabilistically assessing the presence of sleep spindles in EEG signals. We use the intuitively appealing continuous wavelet transform (CWT) with a Morlet basis function, identifying regions of interest where the power of the CWT coefficients corresponding to the frequencies of spindles (11–16 Hz) is large. The potential for assessing the signal segment as a spindle is refined using local weighted smoothing techniques. We evaluate our findings on two databases: the MASS database comprising 19 healthy controls and the DREAMS sleep spindle database comprising eight participants diagnosed with various sleep pathologies. We demonstrate that we can replicate the experts' sleep spindles assessment accurately in both databases (MASS database: sensitivity: 84%, specificity: 90%, false discovery rate 83%, DREAMS database: sensitivity: 76%, specificity: 92%, false discovery rate: 67%), outperforming six competing automatic sleep spindle detection algorithms in terms of correctly replicating the experts' assessment of detected spindles.

## Introduction

Sleep spindles are characteristic oscillatory patterns of brain activity which can be visually detected in human electroencephalography (EEG) signals. These transient patterns are typically portrayed as nearly sinusoidal waxing and waning waveforms with a characteristic frequency profile of 11–16 Hz [formerly this range was narrowed between 12 and 14 Hz in the Rechtschaffen and Kales criteria (Rechtschaffen and Kales, 1968), and different research labs might use slightly different frequency ranges] (Iber et al., 2007; Kryger et al., 2010). Interestingly, although seep spindles exhibit substantially varying characteristics (amplitude, duration, density) in the population, they are fairly stable for individuals (Werth et al., 1997). Spindles are generated in the thalamus, and contemporary evidence suggests they can be classified into slow spindles (11–13 Hz) and fast spindles (13–16 Hz), which are believed to regulate different activation patterns (DeGennaro and Ferrara, 2003).

The presence of sleep spindles is one of the hallmarks for determining stage 2 (S2) in the *hypnogram*, which provides an overall representation of sleep structure successively assigning short signal segments (known as *epochs*, usually of 30 s duration) to one of five sleep stages (Iber et al., 2007). They have been associated with various higher cognitive processes in particular memory (Tamminen et al., 2010), but also learning performance (Schmidt et al., 2006) and skill performance (Astill et al., 2015). Moreover, there is a growing body of research literature highlighting their potential as biomarkers: a number of studies have reported clinically significant differences in spindle characteristics for a range of neurological disorders (Ferrarelli et al., 2007; Wamsley et al., 2012; Christensen et al., 2014).

The gold standard for the determination of sleep spindles has traditionally been achieved through visual inspection of the EEG by sleep physiology experts. Despite the best attempts of experts to standardize protocols, expert-based assessments rely on expensive human resources, depend on the rater's experience and level of expertise, are laborious and prone to errors due to fatigue, and by nature cannot scale to handle very large datasets. As with all cases where the gold standard is set by *subjective* assessments of trained experts, there can always be an argument that an automated algorithmic process could provide an alternative, often sufficiently accurate, robust, scalable, replicable, cost-effective, and objective mode to achieve the aim; indicative studies highlighting these concepts include Grove and Meehl (1996), Seshadrinathan et al. (2010), and Tsanas (2012) amongst many others. At the very least, the development of algorithmic tools can facilitate and expedite the work of trained experts particularly due to the sheer amount of the growing availability of massive datasets.

There are several approaches that have been proposed to tackle the problem of automatic sleep spindle detection. The majority of the proposed algorithms rely on a time-frequency analysis. In all cases, a major hurdle is the determination of appropriate thresholds, which may need to be optimized for each individual. Unfortunately, it is difficult to define universally applicable thresholds due to the large variability in spindle characteristics amongst individuals (Werth et al., 1997). Frequently, the setting of these thresholds for many algorithms require prior hypnogram assessment, and subsequent focusing only on stage 2 sleep (Mölle et al., 2002; Wamsley et al., 2012) or Non Rapid Eye Movement (NREM) sleep (Ferrarelli et al., 2007; Martin et al., 2013). However, we argue that all these approaches are quite restrictive, particularly because in practice we want to completely automate the EEG signal processing task without requiring prior hypnogram assessment by experts. Detecting spindles might be the end goal in one application, but could also be used to guide automated sleep staging assessment. Another generic approach for many algorithms is attempting to determine the presence of spindles by successively searching over pre-defined short windowed EEG segments [typically 1 s, e.g., see Huupponen et al. (2007), although some approaches rely on the detection of spindles in the more traditional 30-s epochs used in hypnogram assessment]. A major limitation with this approach is that one needs to specify a small signal segment to assess whether a spindle occurred within that segment and loosely approximate the spindle onset and offset.

Recently Wendt et al. (2012) introduced a fusion approach to detect spindles applying their sleep detection algorithm on two EEG channels (central and occipital). However, spindles are known to occur locally (Kryger et al., 2010) and hence there is no guarantee that both the central and occipital deflections will identify the spindle; furthermore, this complicates the practical task of spindle assessment by imposing the requirement that additional recordings are available (ideally a single channel would be sufficient for detecting spindles locally). It should be noted that localized sleep can occur, and therefore a single channel cannot reveal the overall sleep structure for the entire brain. In practice we want to focus on specific brain areas, detecting spindles *locally*, e.g., at the central regions where the spindle density is maximal (Kryger et al., 2010); some interesting recent work has focused on spindle propagation (O'Reilly and Nielsen, 2014b).

One of the simplest algorithmic approaches for detecting spindles is to band-pass the EEG signal and assess the presence of spindles by setting an appropriate (relative) threshold on the amplitude of the band-passed version of the signal (Schimicek et al., 1994), which is both sensible and remains topical to this day at least as a benchmark. Similarly, the ubiquitous Fourier Transform (FT) has been investigated in this application (Huupponen et al., 2007). However, there are inherent limitations of the FT in that it implicitly assumes a periodic signal, and also that it requires a sufficiently adequate number of samples for the spectrum estimation; in practice this sets a minimum requirement of about 1 s signal segment (Pardey et al., 1996). In turn, this means that with FT it is fundamentally impossible to correctly determine the spindle onset and offsets accurately as highlighted previously. Wavelet analysis is particularly suitable for analyzing non-stationary signals (such as the EEG), thus overcoming certain shortcomings of the traditional spectral analysis with the FT, and hence has justifiably attracted interest recently in the spindle detection domain (Sitnikova et al., 2009; Wamsley et al., 2012).

This study extends the methodology of recent approaches using the Continuous Wavelet Transform (CWT) with Morlet basis functions (Sitnikova et al., 2009; Wamsley et al., 2012). The Morlet wavelet has been widely used in many practical applications because it has the desirable property that it minimizes the product of the wavelet's time and frequency spreads; hence it optimizes the time-frequency resolution (Addison, 2002). The main novelty of this work lies in the processing of the relative normalized power of the CWT coefficients to determine spindle candidates. Whereas previous studies computed the moving average of the power of the CWT coefficients to detect spindles directly, we first rank the CWT coefficients in terms of their normalized power at each time instant. Then, we compute the instantaneous ratio of the CWT coefficients falling within the scale spindle range (corresponding to the standard 11–16 Hz frequency range) over the top 10 ranked CWT coefficients. This ratio denotes the “instantaneous strength” of detecting a spindle, which is subsequently processed with weighted moving average methods to detect spindles. The proposed algorithm overcomes several shortcomings of competing algorithms: (a) it does not require processing successive small (e.g., 1 s) signal segments which blur the determination of true onset and offset of spindles (instead the algorithm works directly the entire signal), (b) it does not require prior hypnogram assessment, (c) it uses a single EEG lead. Moreover, using the proposed algorithm we can determine the frequency variation contour as a function of time within each spindle: these features may have clinical relevance, a fact which is often overlooked by contemporary competing approaches (for example, FT-based approaches cannot readily provide this information).

## Materials and methods

This section summarizes the dataset used in this study, summarizes some of the previously published algorithms against which the new sleep spindle detection algorithm developed in this study is benchmarked, and outlines the evaluation criteria for assessing the performance of the algorithms.

### Data

We used two publicly available databases in this study.

The first database was collected during the DREAMS project (Devuyst et al., 2011), which aimed to provide a platform to assist assessment of automatic detection algorithms. The sleep spindles database contains recordings from eight participants with diverse sleep pathologies (dysomnia, restless legs syndrome, insomnia, apnoea/hypopnoea syndrome). Two EOG channels (P8-A1, P18-A1), three EEG channels (CZ-A1 or C3-A1, FP1-A1, and O1-A1) and one submental EMG channel were recorded, using a sampling frequency of 200 Hz (six signals), 100 Hz (one signal), or 50 Hz (one signal). A segment of 30 min of a central EEG channel (C3-A1 or Cz-A1) was extracted from each whole-night recording, and two experts have independently annotated the presence of sleep spindles. The second expert has only annotated six out of the eight recordings, and has not provided the exact duration of the assessed spindles (hence, it was all assigned to be 1 s in duration). Although the hypnograms (according to standard Rechtschaffen and Kales criteria) were available, these were not used in the assessment of the spindles by the experts. The dataset along with additional information is publicly available from: http://www.tcts.fpms.ac.be/~devuyst/Databases/DatabaseSpindles/.

The second database was collected as part of a large project looking into sleep, the Montreal Archive of Sleep Studies (MASS) (O'Reilly et al., 2014a). It contains overnight PSG recordings from 19 healthy controls: specifically, electroencephalography (EEG) montage of 19 channels, 4 electro-oculography (EOG), electromyography (EMG), electrocardiography (ECG), and respiratory signals. The EEG signals were sampled at 256 Hz. The database was annotated independently by two experts for sleep spindles. The second expert has only annotated 15 out of the 19 signals for sleep spindles. Hypnograms (according to standard Rechtschaffen and Kales criteria) were also made available. For further details see O'Reilly et al. (2014a). The dataset became available to the authors of this study *after* the development of the algorithms and the original submission of the manuscript; we deliberately decided not to further fine-tune the original algorithms developed using the DREAMS data to guide the sleep spindle estimation process, in order not to bias the presented findings in any way. The dataset can be accessed from: http://www.ceams-carsm.ca/en/MASS.

In all cases, the EEG signals were resampled at 100 Hz.

### Methods

Before delving into the details of the sleep spindle detection algorithms, it is useful to revisit the definition of spindles, and visualize some examples annotated by experts in order to motivate the subsequent algorithmic development. According to the latest recommendation of the AASM Manual for the scoring of sleep, a spindle is defined as “*a train of distinct waves with frequency 11–16 Hz (most commonly 12–14 Hz) with a duration ≥0.5 s, usually maximal in amplitude in the central derivations*.” (Iber et al., 2007). The spindle frequency range is nowadays generally accepted to be 11–16 Hz, but the range over which researchers focus may vary slightly depending on the research lab, e.g., 10.5–16 Hz (Huupponen et al., 2007), or 12–15 Hz (Ferrarelli et al., 2007); the standard reference book “Principles of Sleep Medicine” quotes the range 10–15 Hz (Kryger et al., 2010). We note there is no formal recommendation for the use of amplitude thresholds to detect a spindle, although many researchers have explicitly used amplitude criteria in their algorithmic implementations (Devuyst et al., 2011; Wamsley et al., 2012). Also, many researchers have relaxed the requirement of the minimum spindle duration, e.g., 0.4 s (Wamsley et al., 2012) or even as low 0.3 s instead (Warby et al., 2014). In practice, most spindles are typically around 0.5–1.5 s (very occasionally might be over 2 s), and typically most researchers impose a maximum length constraint (typically 3 s, e.g., Warby et al., 2014) in their algorithmic approaches.

**Figure 1:**
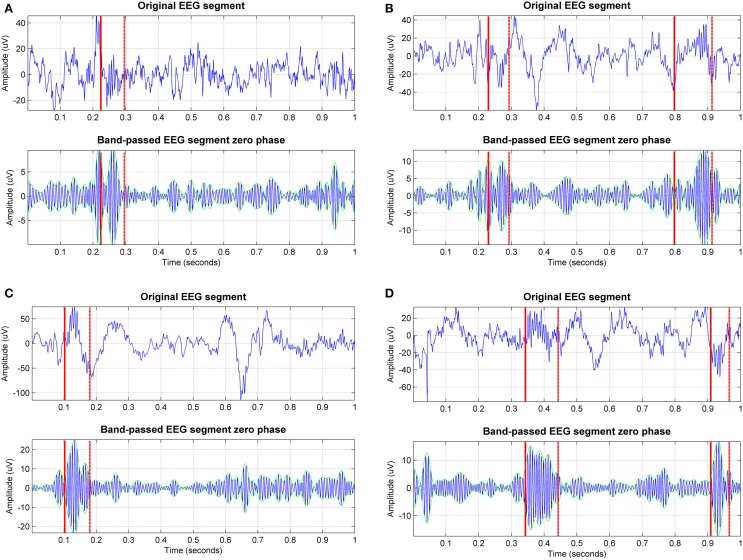
**Exemplary sleep spindles annotated by one of the experts for one of the EEG signals in the DREAMS sleep spindles database (the sampling frequency of the signal is 100 Hz)**. We can visually appreciate the wide variability of sleep spindle characteristics within the same EEG signal. Both the original signal segment and the band-passed (11–16 Hz) version of the signal segment are presented to assist visualization. The solid red line indicates the start of the spindle and the dashed line indicates the end; the green lines indicate the envelope of the signal. In practice, some experts use both the signal and the band-passed version of the signal to assess the presence of spindles.

Sleep textbooks often depict sleep spindles as waxing and waning, nearly sinusoidal waveforms; however, in practice spindle waveforms are markedly noisy, exhibiting diverse characteristics. Figure 1 illustrates some spindles detected by experts for the same signal in the DREAMS sleep spindle database (Devuyst et al., 2011). It is striking that all these transient waveforms (stemming from the same EEG recording and being only a few seconds or minutes apart) display such widely varying features (for example compare the peak-to-peak amplitudes). Nevertheless, all these illustrative examples are considered true spindles according to at least one of the two experts and set the ground truth against which all automated sleep spindle detection algorithms are benchmarked. For each signal we also present its band-passed version at the spindle frequency range. Following visual inspection of these plots, we can postulate that amplitude may be a misleading criterion to assess automatically the presence of spindles; on the other hand, the presence of the spindle appears to be more consistent when also observing the band-pass version of the signals. This exploratory step may assist in the motivation and understanding of the sleep detection algorithms which are presented in the following sections.

#### Contemporary sleep spindle detection algorithms

For simplicity and to conform to the terminology of Warby et al. (2014) we will denote with a_x_ each of the sleep spindle detection algorithms used in this study, where the subscript indicates the corresponding algorithm. In this section we summarize the six spindle detection algorithms used in Warby et al. (2014) (denoted here with a_1_–a_6_), and in the following section we will introduce the new algorithmic approaches. These algorithms (occasionally with slight modifications) have been widely used in a number of studies, and therefore can be considered indicative of the most popular contemporary approaches to automatically detect sleep spindles. We used the Matlab implementations provided by Warby et al. (2014) for a_1_–a_6_ and the description of the algorithms below follows their algorithmic modifications; hence the described algorithms differ slightly in comparison to the original algorithms. Our own algorithms were also implemented in Matlab, and are made freely available on Physionet (www.physionet.org) and the first author's website.

#### Algorithm a_1_, Bódizs' average amplitude spectrum

The first algorithm, a_1_, is due to Bódizs et al. (2009), and attempts to tackle the problem of intra-subject variability in terms of EEG characteristics by incorporating subject-specific information (hence building upon the findings of Werth et al. (1997) that the variability of the spindle characteristics is low for each individual). The algorithm detects spindles in customized frequency ranges (identifying slow and fast spindles) using the average amplitude spectrum of NREM sleep using epochs of 4 s. The decision to evaluate the presence of a spindle is based on the amplitude threshold in each of the two band-pass regions for slow spindles or fast spindles. The implementation by Warby et al. (2014) used here requires both a central and an occipital EEG channel.

#### Algorithm a_2_, Ferrarelli's band pass and signal envelope algorithm

The second algorithm, a_2_, was proposed by Ferrarelli et al. (2007) and with slight modifications has been used in some recent studies, e.g., Astill et al. (2015). The algorithm applies a band-pass filter (11–15 Hz) to the NREM data (epochs), and the envelope of the resulting signal is subsequently used. An amplitude threshold (threshold_1_) is then set relative to the mean signal amplitude (because different channels exhibit different amplitude profiles). A spindle is marked by first detecting a local maximum in the envelope of the filtered signal above threshold_1_, and its duration is determined by identifying the preceding and following instances when this amplitude falls below a lower threshold (threshold_2_), i.e., detecting the nearest troughs below threshold_2_ (local minima). The slightly different versions of this type of algorithm set threshold_1_ and threshold_2_ slightly differently than the original algorithm, but the essential main idea remains the same.

#### Algorithm a_3_, Mölle's band pass RMS overlapping moving window

The third algorithm, a_3_, was described by Mölle et al. (2002). This algorithm is also band-pass filtering the NREM data at the spindle frequency range (12–15 Hz), and subsequently computes the Root Mean Squared (RMS) value of the filtered data over a short-frame overlapping (50%) moving window of 100 ms. Then, spindles are determined only on the data from sleep stage 2 depending on whether the RMS value exceeds an amplitude threshold (set at 1.5 times the standard deviation of the band-pass filtered signal) and the duration is within the acceptable spindle limits (0.3–3 s).

#### Algorithm a_4_, Martin's band pass RMS percentile moving window

The fourth algorithm, a_4_, by Martin et al. (2013) is conceptually very similar to a_3_. It differs from a_3_ in terms of the spindle frequency range used (11–15 Hz) for the band-pass filter, the use of a non-overlapping time window (25 ms) to compute the RMS values, and the threshold for detecting the spindle which is set to be the 95th percentile of the RMS signal.

#### Algorithm a_5_, Wamsley's CWT moving average

The fifth algorithm, a_5_, was developed by Wamsley et al. (2012). Contrary to the algorithms described so far, this algorithm is based on the CWT, which has some desirable properties for analyzing EEG signals as discussed previously. The algorithm relies on prior hypnogram assessment and attempts to detect spindles during stage 2. The signal is transformed into the wavelet domain using the complex Morlet wavelet basis function. The Morlet scales corresponding approximately to the pseudo-frequencies of interest (10–16 Hz) were used, and the moving average of the coefficients using a 100 ms sliding window was computed; when it exceeded a threshold for a minimum of 0.3 s a spindle was registered. The threshold was set using only the amplitude of epochs assessed as stage 2 by experts.

#### Algorithm a_6_, Wendt's two-channel band pass and signal envelope combination

The sixth algorithm, a_6_, was developed by Wendt et al. (2012). This algorithm is conceptually similar to a_2_, the main difference is that the boundaries for the spindle detection are determined using local extrema of the signal envelope and its rate of change, whereas a_2_ relied on local minima. A further difference is that both a central and an occipital EEG channels are used in the band 11–16 Hz, and the spindle detection is a result of the combination of the two different sets of envelopes.

Recently, Warby et al. (2014) applied the six algorithms described so far in a large private database with sleep spindles from 110 healthy controls, and reported that the best algorithm in terms of accurately detecting spindles and minimizing false detections was a_5_, closely followed by a_4_. We note that all six algorithms described so far (a_1_–a_6_) rely on prior hypnogram assessment, which was provided given that the sleep stages assessed by experts was available for this database. We note that this fact effectively places competing algorithms which do not have access to hypnogram information at a disadvantage when it comes to direct algorithmic performance comparisons. The following new algorithms (a_7_–a_8_) do not rely on prior sleep staging information, but we aim to demonstrate that the new algorithms are nevertheless very competitive.

#### Novel sleep spindle detection algorithms

We have already highlighted the intuitively appealing features of the CWT for analyzing EEG signals due to its time-frequency localization properties, and the fact that it does not make assumptions regarding signal periodicity. Exploring the data by visual inspection of the true spindles (see Figure 1) seems to indicate that amplitude-based characteristics may be misleading (this is also implicit in the AASM criteria where no amplitude recommendation is made when assessing spindles); hence the primary focus of the developed algorithms is on the frequency content of the signal. Strictly speaking, we work directly with the CWT scales which correspond to the (pseudo)frequencies of interest (11–16 Hz). We defined 131 Morlet scales with a resolution of 0.1 in the range 2–15 (corresponding pseudo-frequencies: 5.4–40.6 Hz), which led to 24 scales lying within the spindle scale range. There is a non-linear mapping of the scales to their corresponding pseudo-frequencies, which is a function of the wavelet basis function and the sampling frequency of the signal. For the Morlet wavelet with a signal sampling frequency of 100 Hz, the scales of interest (*spindle scale range*) are 5.1–7.4. We used a lower threshold of pseudo-frequency at 5.4 Hz above which we try to assess the probability of having a spindle so as to avoid challenging settings of spindles occurring on the background of large-amplitude slow oscillations (the delta frequency range, 1–4 Hz). Conceptually the starting basis of the proposed algorithms is similar to the study by Wamsley et al. (2012) (algorithm a_5_), who subsequently thresholded the CWT coefficients at the spindle frequency range using a moving average of 100 ms sliding window. What distinguishes the algorithms proposed in this study compared to previous algorithms using the CWT is the different processing of the extracted Morlet CWT coefficients and the fact that we do not rely on expert-based hypnogram (in particular determining sleep stage 2) assessment.

**Figure 2:**
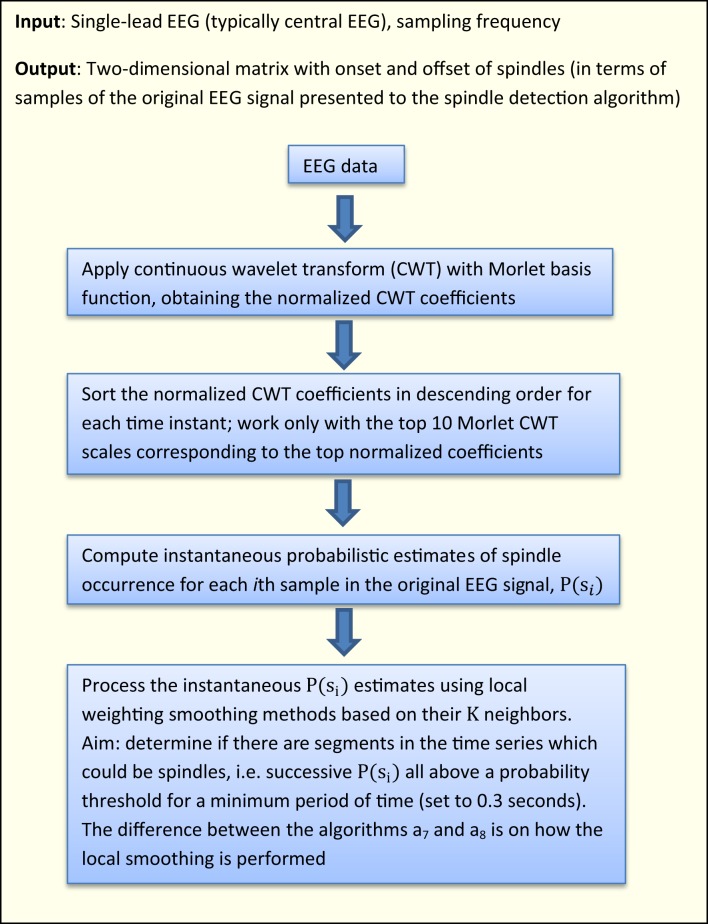
**Flowchart of the proposed algorithms in this study**.

Figure 2 presents a high-level flowchart of the two new algorithms introduced in this study. All sleep spindle detection algorithms developed in the research literature have some free parameters (typically these are some thresholds, e.g., on amplitude values). Similarly, the proposed algorithms in this study rely on a number of free parameters which need to be optimized: the chosen values were determined by testing on random subsamples of the training data so that regions of relative stability were found; exhaustive searches over the parameter space were not possible due to the size of the data set. We deliberately decided not to pursue rigorous optimization of these parameter values, in order to avoid overfitting the characteristics in the DREAMS database (effectively this would be training and testing on the same data). It is likely that the parameter values chosen could benefit from further refinement to optimize the outputs of the proposed algorithms, but a larger database would be needed.

#### Algorithm a_7_, CWT instantaneous probabilistic estimate with moving averaging

The algorithm a_7_, uses the following steps after the computation of the CWT coefficients:

- Computes the normalized percentage power of the CWT coefficients (henceforth referred to as *normalized coefficients*).
- Sorts the normalized coefficients in descending order at each time instant and works on the top 10 Morlet CWT scales corresponding to the top normalized coefficients (thus resulting in a matrix of size *number of signal samples* × 10).
- Computes instantaneous probabilistic estimate of spindle occurrence at the spindle scale range using the following algorithmic expression: 
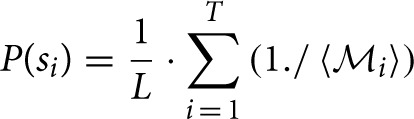
 where P(s*_i_*) denotes the probability of having a spindle at a given sample *i*, *T* is the cardinality of the top 10 scales corresponding to the sorted top 10 CWT normalized coefficients at instant *i* coinciding with the spindle scale range (i.e., for each sample *i*, we find how many of the top 10 sorted scales corresponding to the normalized coefficients match the scales in the spindle scale range), 〈
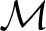
*_i_*〉 contains the positions of the detected scales intersecting with the spindle scale range in the 10-element vector and the operator “./” denotes element-wise division. The value P(s*_i_*) effectively expresses the confidence that the sample *i* is part of a spindle (the higher the value, the more likely this sample may be part a spindle). The underlying concept is that if a sufficiently large number of successive samples (corresponding to some minimum time duration to be defined) have large probabilities denoting spindles, then that sequence will be denoted as a spindle. Effectively, we determine how many of the top 10 sorted scales matched the spindle scale range, and weigh these scales based on where they feature in the list with the instantaneous top 10 scales. If none of the sorted top 10 scales overlapped with the spindle scale range then P(s*_i_*) is zero. *L* denotes a normalization constant factor which was computed as $L=\sum_{i\;=\;1}^{T}\left(1./\langle 1\ldots 10\rangle \right)$.
- Now, we need to smooth the instantaneous P(s*_i_*) estimates based on their *K* neighbors {*P*(*s_i_*_−*K*/2_) … *P*(*s_i_*_+*K*/2_)} to determine whether some EEG segments (regions) of arbitrary length within some duration boundaries (here 0.5–1.5 s) correspond to a spindle. Essentially, we have scales corresponding to the spindle scale frequency and we want to smooth neighboring regions to decide whether these are above the minimum duration threshold (in practice we very rarely have *all* consecutive samples in a spindle exhibiting large proportion of the scales belonging to the spindle scale range). Conceptually, this is similar to the concept that Wamsley et al. (2012) used, smoothing the data using a moving average of 0.1 s. Similarly, we used a moving average filter of 0.1 s to obtain the P*_smooth_*(s*_i_*).
- It is possible that certain P (s*_i_* < P*_smooth_*(s*_i_*) and we want to encourage relative large values to maximize the probability of detecting true spindles; hence we applied a final check: P*_final_* (s*_i_*) = max_∀*i*_ (P*_smooth_* (s*_i_*), P (s*_i_*)).
- The candidate spindle instances (as a first pass) were detected at those samples when P*_final_* (s*_i_*) > 0.3 (for as many successive samples as the threshold remains valid). We remark this threshold (and all free parameters in this spindle detection algorithm such as number of top scales to investigate and *K*) were not rigorously optimized to avoid over-fitting the database used in this study. Instead we have attempted to determine “good” parameter values, which may be refined if presented with additional databases which will assist in properly optimizing the values of the free parameters.
- Finally, we need to group together regions which contain series of samples with high probabilities of denoting spindles. This was achieved using flags to denote if successive regions containing candidate spindles would group in terms of their proximity, average probabilistic estimate of having a spindle in a region defined between samples (*i*_1_, …, *i*_2_) {P(s_*i*_1__) … P(s_*i*_2__)} and the duration of the candidate spindle. Specifically, we grouped successive candidate spindles in the following cases:
  - The duration between successive spindles was less than 0.3 s, and both successive spindles exhibited average probabilistic strength above a threshold, i.e., both spindles appeared to be very likely true spindles: $\left(\frac{1}{i_{2}-i_{1}}\cdot \sum_{i\;=\;i_{1}}^{i_{2}}\text{P}\left({\text{s}}_{i}\right)\right)>0.7$, and the duration of both successive spindles was at least 0.1 s (case: “strong” spindles).
  - The duration between successive spindles was less than 0.3 s and both successive spindles exhibited average probabilistic strength: $\left(\frac{1}{i_{2}-i_{1}}\cdot \sum_{i\;=\;i_{1}}^{i_{2}}\text{P}\left({\text{s}}_{i}\right)\right)>0.6$ and both were at least 0.3 s long (case: “long spindles”).

#### Algorithm a_8_, CWT instantaneous probabilistic estimate with distance and amplitude weighted averaging

The algorithm a_8_, is very similar to a_7_. The difference lies in how we process the instantaneous probability spindle estimates P(s*_i_*) to affect neighboring P(s*_j_*) values. That is, the first steps (a)–(c) are identical, and step (d) processes the computed P(s*_i_*) using the exponential weighted moving average concept (instead of moving average). The underlying idea is that we want to update P(s*_i_*) values depending on their neighboring P(s*_j_*) values as a weighted function of their distances and a weighted function of their magnitude (which is weighted exponentially to promote EEG regions where instantaneous P(s*_i_*) estimates are large). Specifically, step (d) now becomes:

(d) We used smoothing over 0.2 s, linearly scaling the effect of samples P(s*_j_*) on P(s*_i_*) as a function of their distance from P(s*_i_*), i.e., ${\left\{w_{t}\right\}}_{t=-10,\;t\neq 0}^{10}=\frac{1}{\left|t\right|}\cdot \text{P}({\text{s}}_{i\;+\;t})$. In order to augment the effect of large P(s*_i_*) values (which denote great confidence that the sample *i* is part of a spindle) we exponentiated these values. Overall, conceptually it is similar to using an exponential weighted moving average approach. Algorithmically this is expressed as:
  $$\begin{array}{l} {\text{P}}_{smooth}\left({\text{s}}_{i}\right)=\lceil \text{P}\left({\text{s}}_{i}\right)+\frac{1}{\sum_{t\;=\;-10,\;t\;\neq \;0}^{10}w_{t}}\\ \;\cdot \sum_{t\;=\;-10,\;t\;\neq \;0}^{10}\left(\exp\left(\text{P}\left({\text{s}}_{i\;+\;t}\right)\right)-1\right).*w_{t}\rceil \end{array}$$
  where the notation ⌈·⌉ denotes that the value is upper bounded to be 1, and the notation “.^*^” denotes element wise multiplication. The subsequent steps (e)–(g) are identical to a_7_ to detect a spindle. We remark that a_8_ is by design heavily weighting regions where there is a possibility of observing a spindle, but these regions will likely contain many cases which are not likely to be spindles.

#### Evaluation of sleep spindle detection algorithms

Both the DREAMS sleep spindles database and the MASS database have been annotated by two experts. Given the large inter-rater variability (e.g., for the DREAMS database the first rater has marked 289 spindles whereas the second rater has marked 409 spindles), there are two approaches to determine the ground truth. One approach is to only consider cases where both experts agree, an approach used previously for the DREAMS database by other researchers (Devuyst et al., 2011; Nonclercq et al., 2013). However, this biases the results, because one might argue that cases where both experts agree may denote “easily detectable” spindles; hence in this study we used all assessments by both experts, removing one of the double entries (in those cases where both experts agreed, in the DREAMS database we removed the assessment by the second expert because only the first expert had also provided the duration of the assessed spindle).

Each of the sleep spindle algorithms used in this study results in estimates summarized in the format *N*× 2, where *N* denotes the number of detected spindles for each EEG signal: the first column contains the estimated onset, and the second column the spindle duration. This facilitates direct comparison with the ground truth which is in the same format. In order to assess the performance and fairly compare all algorithms, we used the following commonly used metrics:

- True Positive Rate (TPR) (%), also known as *sensitivity*: TPR = TP/(TP + FN) (is the proportion of spindles assessed by experts correctly identified by an algorithm, ideally we want this to be 100%).
- True Negative Rate (TNR) (%), also known as *specificity*: TNR = TN/(TN + FP) (is the proportion of non-spindles assessed by experts correctly identified by an algorithm, ideally we want this to be 100%).

Specificity is also the complement of the False Positive Rate (FPR), defined as FPR = FP/(FP + TN): specificity = 100—FPR.

(c) False Discovery Rate (FDR): FDR = FP/(TP + FP).
(d) Cohen's kappa coefficient, where: $k=\frac{\frac{TP+TN}{N}-\text{Pr}(e)}{1-\text{Pr}(e)}$, with $\mathrm{Pr}\left(e\right)=\frac{TP+FN}{N}\cdot \frac{TP+FP}{N}+\left(1-\frac{TP+FN}{N}\right)\cdot \left(1-\frac{TP+FP}{N}\right)$, and *N* = *TP* + *FP* + *TN* + *FN*

Cohen's kappa coefficient was originally developed to assess inter-rater agreement, and some researchers suggest it takes into account agreement between raters which could be attributed to chance. Effectively, this implies that when raters are uncertain they guess about their decision, which some researchers have suggested is unlikely in many practical settings. Some of the problems and limitations of Cohen's kappa have been discussed by Gwet (2008); we cautiously include it in this study because some research papers published in the sleep spindle detection literature have used it. We also used and put greater emphasis on the weighted kappa in this study because spindles are rare events in the EEG signal and we wanted to weigh accordingly for spindles correctly detected and spindles missed by the spindle detection algorithms (that is, we set the weight for TP and FN to be 10 times compared to the weight assigned to FP and TN).

(e) Absolute difference in the onset timings between the ground truth and the estimated onset.

where True Positive (TP) denotes agreement between the algorithm and the ground truth about the detection of a spindle, False Negative (FN) denotes a true spindle as assessed by the experts which was missed by the algorithm, False Positive (FP) when the algorithm detected a spindle that was not assessed as a spindle by the experts, and True Negative (TN) was defined as in Devuyst et al. (2011): TN = signal duration in seconds − FP − TP − FN. We assess a true positive when the absolute difference between the onset of the ground truth and the estimated spindle onset by the algorithm is less than 0.5 s. Other studies have used different, less stringent definitions to assess whether an algorithm has matched the expert's assessment in correctly detecting a spindle. Some studies assess whether a spindle was detected within a sliding pre-specified time-interval (epoch), e.g., Duman et al. (2009), however this does not assess directly the accuracy in determining the spindle onset. Other studies, e.g., Nonclercq et al. (2013), consider than an algorithm has correctly detected a spindle if there was *any* overlap between the duration of the estimated spindle and the true spindle duration. However, this may positively bias sleep detection algorithms which provide spindle estimates with large durations.

## Results

### Evaluation of the spindle detection algorithms on the DREAMS sleep spindles database

Tables 1–3 summarize the performance of the sleep spindle detection algorithms used in this study for each of the eight signals. Ideally, a good algorithm exhibits large sensitivity and specificity, and low false discovery rate.

**Table 1:** **Sensitivity (%) of the spindle detection algorithms across the eight EEG signals (higher values indicate better performance)**.

	**Signal_1_**	**Signal_2_**	**Signal_3_**	**Signal_4_**	**Signal_5_**	**Signal_6_**	**Signal_7_**	**Signal_8_**	**Mean ± std**
a_1_	70.6	56.6	53.3	40.6	45.6	78.6	27.8	75	56.0±17.8
a_2_	14	3.90	11.1	9.40	20.4	29.1	16.7	10.4	14.4±7.7
a_3_	86.7	68.8	**84.4**	42.2	95.1	**91.5**	77.8	75	77.7±16.8
a_4_	46.7	63.6	77.3	32.8	63.1	68.4	61.1	50	57.9±14.0
a_5_	12.5	49.4	**84.4**	31.3	15.5	64.1	55.6	47.9	45.1±24.4
a_6_	79.3	**85.7**	77.8	45.3	81.6	81.2	72.2	83.3	75.8±12.9
a_7_	84.4	80.5	73.3	65.6	70.9	66.7	**88.9**	77.1	75.9±8.3
a_8_	**89**	80.5	82.2	68.8	**96.1**	88	77.8	**83.3**	**83.2±8.2**

*The best performing algorithm for each case appears in bold*.

**Table 2:** **Specificity (%) of the spindle detection algorithms across the eight EEG signals (higher values indicate better performance)**.

	**Signal_1_**	**Signal_2_**	**Signal_3_**	**Signal_4_**	**Signal_5_**	**Signal_6_**	**Signal_7_**	**Signal_8_**	**Mean ± std**
a_1_	85	79.8	83.9	82.9	82.6	85.2	80.7	79.1	82.4±2.3
a_2_	**99.6**	**100**	**99.6**	**98.8**	**99.2**	**99.4**	**98.9**	**99.1**	**99.3±0.4**
a_3_	91.1	97.6	75.1	92.7	88.5	77.8	89.1	39	81.4±18.7
a_4_	98.5	98.3	97	96.5	98.2	98.6	95.6	94.3	97.1±1.6
a_5_	99.8	99.2	96.1	96.3	99.6	98.8	97.1	96.1	97.9±1.6
a_6_	86.6	67	87	87.1	91.1	92.5	82	79.5	84.1±8.1
a_7_	94.6	93.4	94.5	87.3	95.5	97.3	94.1	78.1	91.8±6.3
a_8_	78.6	76.3	77.8	68.1	80.9	86.6	75.5	55.7	74.9±9.4

*The best performing algorithm for each case appears in bold*.

**Table 3:** **False discovery rate (%) of the spindle detection algorithms across the eight EEG signals (lower values indicate better performance)**.

	**Signal_1_**	**Signal_2_**	**Signal_3_**	**Signal_4_**	**Signal_5_**	**Signal_6_**	**Signal_7_**	**Signal_8_**	**Mean ± std**
a_1_	72.3	89	92.2	91.9	86.3	73	98.6	91.1	86.8±9.4
a_2_	26.9	**0**	**58.3**	77.8	38.2	22.7	87	75	**48.2±31.0**
a_3_	56	44.2	92	82.4	66.7	77.7	93.3	96.7	76.1±19.0
a_4_	28.4	38	60.5	**74.4**	32.3	23.1	87.6	80.6	53.1±25.7
a_5_	**19**	25.5	64.2	76.5	**27.3**	**21.9**	**83.9**	**74.7**	49.1±28.1
a_6_	67.6	89.6	86.7	88.5	64.3	57.2	96.1	90	80.0±14.6
a_7_	44.1	64.6	74.4	84	51.3	37.1	86.9	91.2	66.7±20.7
a_8_	74.6	86.8	91.3	92.6	76.6	68.6	96.9	95.1	85.3±10.6

*The best performing algorithm for each case appears in bold*.

We observe relatively large deviations in the performance of the sleep spindle detection algorithms across the eight signals. Overall, the new algorithm a_7_ exhibits large sensitivity and specificity. The more complicated new algorithm a_8_ can accurately detect more spindles than the competing approaches including a_7_ (large sensitivity), at the cost of decreased specificity and increased false discovery rate. We have also evaluated the absolute difference in the onset timings between the ground truth and the estimated onset: this was fairly consistent amongst the algorithms with a mean absolute difference in onset timings ranging between 0.15 and 0.2 s and the standard deviation ranging between 0.11 and 0.15 s. Overall, all algorithms performed similarly with respect to correctly detecting onset spindle timing. We have emphasized that Cohen's kappa suffers from certain limitations (Gwet, 2008) and we use it here cautiously simply to facilitate comparisons with other studies in the research literature. Specifically the (unweighted) Cohen kappa was (mean ± standard deviation): a_1_ = 0.15 ± 0.12, a_2_ = 0.19 ± 0.11, a_3_ = 0.29 ± 0.22, a_4_ = 0.46 ± 0.20, a_5_ = 0.37 ± 0.19, a_6_ = 0.25 ± 0.18, a_7_ = 0.40 ± 0.20, a_8_ = 0.18 ±0.14.

### Evaluation of the spindle detection algorithms on the MASS database

We have also evaluated the performance of all eight algorithms in terms of correctly detecting the sleep spindles in the MASS database. The results are summarized in Table 4. Interestingly, the findings in terms of sensitivity, specificity, and FDR are similar across the two databases used in this study. The algorithm a_7_ outperforms the competing approaches in terms of sensitivity whilst being very competitive in terms of specificity. As indicated previously, we prefer the weighted Cohen kappa (see Table 4) penalizing more severely missed true spindles compared to false positives. Nevertheless, to facilitate direct comparisons with the research literature the unweighted Cohen kappa for the algorithms is also reported (mean ± standard deviation): a_1_ = 0.20 ± 0.11, a_2_ = 0.22 ± 0.04, a_3_ = 0.28 ± 0.24, a_4_ = 0.51 ± 0.13, a_5_ = 0.38 ± 0.18, a_6_ = 0.37 ± 0.18, a_7_ = 0.24 ± 0.12, a_8_ = 0.16 ± 0.09.

**Table 4:** **Summary of automated spindle detection results in the research literature and in this study**.

**Study**	**Spindle assessment**				**Participants and data collected**	**Database**	**Algorithm requires hypnogram**	**Spindle detector TP evaluation**
	**Sensitivity (%)**	**Specificity (%)**	**FDR (%)**	**Weighted Cohen kappa**				
Schonwald et al., 2006	81.2	81.2	N/R	N/R	9 healthy adults, extracted 24 segments from each subject using 20 s epochs, removed epochs with artifacts	Private (*N* = 9)	Yes	Second-by-second analysis
Huupponen et al., 2007	70.0	98.6	32	N/R	12 healthy adults, entire night recordings	Private (*N* = 12)	Yes	The absolute difference between the detected spindle onset and the spindle onset determined by the experts was less than 0.5 s.
Causa et al., 2010	88.2	89.7	11.9	N/R	56 healthy children overnight recordings, 27 recordings used for training, 10 recordings for validation, and 19 for testing performance	Private (*N* = 56)	No	At least 75% spindle duration overlap between detected and expert assessed spindle
Warby et al. (2014) applying a_1_	74	81	89	N/R	110 healthy adults, (4 min of artifact-free stage 2 sleep from 100 subjects and ~38 min of stage 2 sleep from 10 subjects)	Private (*N* = 110)	Yes	At least 20% spindle duration overlap between detected and expert assessed spindle
Warby et al. (2014) applying a_2_	17	99	48	N/R	See above entry	Private (*N* = 110)	Yes	See above entry
Warby et al. (2014) applying a_3_	71	81	89	N/R	See above entry	Private (*N* = 110)	Yes	See above entry
Warby et al. (2014) applying a_4_	43	98	58	N/R	See above entry	Private (*N* = 110)	Yes	See above entry
Warby et al. (2014) applying a_5_	33	99	44	N/R	See above entry	Private (*N* = 110)	Yes	See above entry
Warby et al. (2014) applying a_6_	57	96	70	N/R	See above entry	Private (*N* = 110)	Yes	See above entry
Devuyst et al., 2011	70.2	98.6	N/R	N/R	8 diagnosed with various sleep disorders (30 min segments), two raters for all signals; one rater only for two signals. Use only six signals and only cases where raters agree	DREAMS sleep spindle database (publicly available) (*N* = 6)	No	N/R
Nonclercq et al., 2013	75.1	96.7	N/R	N/R	See above entry	DREAMS (*N* = 6)	No	There is overlap between the duration of the detected spindle and the spindle duration assessed by experts
Present study a_1_	56.0	82.4	86.8	0.37	8 from various sleep disorders (30 min segments), two raters for all signals; one rater only for two signals. Use all eight signals including “difficult” cases where raters do not agree	DREAMS (*N* = 8)	Yes	The absolute difference between the detected spindle onset and the spindle onset determined by the experts was less than 0.5 s
Present study a_2_	14.4	99.3	48.2	0.17	See above entry	DREAMS (*N* = 8)	Yes	See above entry
Present study a_3_	77.7	81.4	76.1	0.55	See above entry	DREAMS (*N* = 8)	Yes	See above entry
Present study a_4_	57.9	97.1	53.1	0.59	See above entry	DREAMS (*N* = 8)	Yes	See above entry
Present study a_5_	45.1	97.9	49.1	0.47	See above entry	DREAMS (*N* = 8)	Yes	See above entry
Present study a_6_	75.8	84.1	80.0	0.55	See above entry	DREAMS (*N* = 8)	Yes	See above entry
Present study a_7_	75.9	91.8	66.7	0.66	See above entry	DREAMS (*N* = 8)	No	See above entry
Present study a_8_	83.2	74.9	85.3	0.50	See above entry	DREAMS (*N* = 8)	No	See above entry
Present study a_1_	65.5	85.1	82.7	0.46	19 overnight PSG from healthy controls; two raters for 15 signals, one rater for four signals	MASS database S2 (publicly available) (*N* = 19)	Yes	See above entry
Present study a_2_	16.5	99.2	49.5	0.20	See above entry	MASS (*N* = 19)	Yes	See above entry
Present study a_3_	73.5	78.2	75.3	0.46	See above entry	MASS (*N* = 19)	Yes	See above entry
Present study a_4_	66.2	97.5	48.1	0.64	See above entry	MASS (*N* = 19)	Yes	See above entry
Present study a_5_	41.3	98.8	45.3	0.43	See above entry	MASS (*N* = 19)	Yes	See above entry
Present study a_6_	73.0	90.5	69.1	0.60	See above entry	MASS (*N* = 19)	Yes	See above entry
Present study a_7_	83.8	90.2	82.6	0.64	See above entry	MASS (*N* = 19)	No	See above entry
Present study a_8_	77.2	76.9	86.5	0.46	See above entry	MASS (*N* = 19)	No	See above entry

*Sensitivity (%) = TP/(TP + FN), Specificity (%) = TN/(TN + FP), False Discovery Rate (FDR) (%) = FP/(TP + FP). TP stands for true positive, TN for true negative, FP for false positive, and FN for false negative. The last column briefly explains the method used to assess how the automatic sleep spindle detector was deemed to succeed in detecting the spindle as registered by the experts. See Section Evaluation of Sleep Spindle Detection Algorithms for more details*.

### Algorithmic comparisons with results reported in the research literature

Many researchers have indicated that it is not easy to directly compare the performance of different algorithms across studies because of the different criteria used to detect spindles and assess the performance of the automated algorithms (Devuyst et al., 2011; Nonclercq et al., 2013). Table 4 attempts to summarize many of these published findings in the research literature as an indicative reference, but we emphasize these results should be cautiously interpreted when comparing algorithms unless they have been tested on the same database using identical criteria to assess performance. Table 5 summarizes the four performance metrics in this study (sensitivity, specificity, FDR, weighted Cohen's kappa) in terms of percentile scores, thus providing a good overview of the overall performance of each algorithm (including their behavior at extremes).

**Table 5:** **Summary of statistics (percentiles) of the performance metrics of the spindle detection algorithms for the DREAMS and MASS databases**.

	**Sensitivity (%)**					**Specificity (%)**					**FDR (%)**					**Weighted Cohen kappa**				
	**5**	**25**	**50**	**75**	**95**	**5**	**25**	**50**	**75**	**95**	**5**	**25**	**50**	**75**	**95**	**5**	**25**	**50**	**75**	**95**
a_1_	27.8	43.1	54.9	72.8	78.6	79.1	80.3	82.8	84.5	85.2	72.3	79.6	90	92.1	98.6	0.06	0.27	0.36	0.51	0.64
	54.1	60.8	65.3	69.3	80.84	82.1	83.7	85.3	86.4	88.4	66.3	76.3	80.6	90.9	97.7	0.22	0.43	0.49	0.54	0.63
a_2_	3.9	9.9	12.6	18.6	29.1	98.8	99.0	99.3	99.6	100	0	24.8	48.3	76.4	87.0	0.05	0.13	0.15	0.23	0.32
	10.9	13.0	14.6	17.5	30.1	98.9	98.9	99.2	99.4	99.6	33.8	41.8	43.9	64.2	67.0	0.12	0.15	0.18	0.20	0.39
a_3_	42.2	71.9	81.1	89.1	95.1	39	76.5	88.8	91.9	97.6	44.2	61.3	80	92.7	96.7	0.08	0.43	0.58	0.75	0.81
	34.5	58.1	81.7	88.8	91.6	39.4	64.2	83.9	93.4	97.0	35.8	58.7	76.8	96	98.7	0	0.10	0.62	0.75	0.82
a_4_	32.8	48.4	62.1	66.0	77.3	94.3	96.1	97.6	98.4	98.6	23.1	30.4	49.2	77.5	87.6	0.36	0.49	0.61	0.69	0.77
	41.2	56.2	64.8	77.6	96.2	95.7	97.2	97.6	98.2	98.7	23.5	33.2	43.7	64.4	88.5	0.40	0.58	0.68	0.73	0.82
a_5_	12.5	23.4	48.7	59.9	84.4	96.1	96.2	97.9	99.4	99.8	19.0	23.7	45.7	75.6	83.9	0.13	0.26	0.54	0.61	0.81
	3.5	24.7	39.6	48.8	91.4	97.1	98.5	98.9	99.5	99.7	20.6	29.8	39.7	58.8	82.0	0.040	0.35	0.43	0.54	0.78
a_6_	45.3	75.0	80.3	82.5	85.7	67.0	80.8	86.8	89.1	92.5	57.2	65.9	87.6	89.8	96.1	0.32	0.40	0.55	0.70	0.75
	52.4	69.8	72.7	76.0	92.79	76.7	85.9	92.8	95.2	97.4	45.7	55.6	66.1	80.7	97.0	0.23	0.60	0.65	0.69	0.74
a_7_	65.6	68.8	75.2	82.5	88.9	78.1	90.4	94.3	95.1	97.3	37.1	47.7	69.5	85.5	91.2	0.46	0.60	0.69	0.72	0.80
	64.7	80.1	86.3	89.6	92.9	83.6	88.1	90.1	94.1	95.9	51.3	81.1	85.7	90.6	92.3	0.49	0.60	0.64	0.70	0.74
a_8_	68.8	79.2	82.8	88.5	96.1	55.7	71.8	77.1	79.8	86.6	68.6	75.6	89.1	93.9	96.9	0.26	0.29	0.50	0.70	0.74
	65.1	72.7	79.3	82.2	87	67.6	72.6	76.2	81.1	86.5	71.0	83.0	86.8	92.2	97.6	0.24	0.36	0.49	0.58	0.63

*The first row for each algorithm a_1_–a_8_ corresponds to the (5,25,50,75,95) percentiles in the DREAMS database, and the second row to the percentiles in the MASS database*.

## Discussion

This study revisited the problem of accurate and automatic detection of sleep spindles using a single EEG channel. We reviewed some indicative and widely used signal processing approaches toward this aim, and highlighted some of the underlying problems. Two new signal processing approaches which are based on the CWT with Morlet basis were proposed and demonstrated to be very competitive against some commonly used algorithms found in the research literature. Interestingly, there was no universally best algorithm for all signals, although a_3_, a_6_, and a_7_ appear to display relatively large sensitivity and specificity scores. We found that the new algorithm a_7_ led to a range of 65.6–88.9% sensitivity scores and a range of 78.1–97.3% specificity scores for the DREAMS database, which compare favorably against competing approaches. The new algorithm a_8_ exhibits higher sensitivity and lower specificity in the DREAMS database, on average, hence it might be more suitable primarily in cases where a human expert will post-process the estimates to determine whether the detected spindles correspond to true spindles. We re-iterate that the DREAMS sleep spindles database used in this study suffers from large inter-rater variability: the first rater has marked 289 spindles whereas the second rater has marked 409 spindles. Hence, the inter-rater agreement is lower than the agreement between raters reported in other studies (Huupponen et al., 2007), which may suggest automatic detection of spindles in this dataset may be challenging.

The original manuscript submission did not include the MASS database and hence the development of the spindle detection algorithm relied only on the DREAMS data. We have deliberately refrained from any additional fine-tuning of a_7_ and a_8_ to optimize performance in the MASS data, which might have potentially improved our reported results on the MASS database. It is reassuring that the proposed algorithms work very well on the MASS data, in particular a_7_. It is also encouraging to see that the results of sensitivity, specificity, FDR and weighted Cohen's kappa are similar across the two databases (see Table 4) for all algorithms: this inspires confidence regarding the objective merits of each algorithm, and may be a good indicator of the performance of the sleep spindle detection algorithms in new, unseen datasets. It is possible that other studies relying on a single database to develop and test their spindle detection algorithms might have over-trained on that particular dataset, so we find the reported findings on the MASS database (truly out-of-sample) to be particularly compelling. Table 5 provides an overall summary of performance of the sleep spindle algorithms on both databases, including extremes (i.e., the algorithms at their worst and at their best) by reporting percentile values. We note that a_7_ in particular is very competitive across the entire range of the distribution of performances, particularly for the MASS database (and interestingly, exhibiting good performance even for the 5th and 25th percentiles, i.e., it is fairly stable across individuals compared to many of the competing algorithms).

For reference purposes we have summarized the findings of multiple sleep spindle studies in the research literature in Table 4. However, direct comparison of findings across studies in this application is not straightforward for a number of reasons: (a) many studies rely solely on data stemming from healthy controls which are arguably easier to analyze than data from pathological cohorts (or process EEG artifact-free data, whereas the DREAMS sleep spindle database used here contains data from various sleep disorders), (b) the criteria for identifying sleep spindles are inconsistent, (c) different research teams use slightly different definitions of spindles, (d) in some cases researchers have only reported the detection accuracy but have not provided details about the number of erroneous detections, therefore making comparison against some conservative approaches (algorithms which aim to minimize the number of falsely reported spindles) unfair. For all these reasons, probably the most efficient and appropriate scientific approach is to apply multiple sleep spindle detection algorithms across multiple datasets and directly compare their performance. Causa et al. (2010) have reported better sensitivity (88.2%) and specificity scores (89.7%) compared to results in other studies (including the current study). However, that study focused only on healthy children, and those findings might not be generalizable to studies focusing on other cohorts (healthy adults, and adults diagnosed with a sleep-related disorder). Two prior studies have focused on the DREAMS sleep spindle database which facilitate comparison of findings: Devuyst et al. (2011) reported sensitivity score 70.2% and specificity score 98.6%. Likewise, Nonclercq et al. (2013) reported sensitivity scores ranging between 65.8 and 82.8% and specificity scores ranging between 96.7 and 98.7% for the first six signals in the database. However, we note that in both studies the authors used as ground truth only those cases where the experts agreed on the first six signals, which potentially biases the results (spindles detected by either one of the raters are probably borderline and more difficult to assess, but on the other hand are probably also more interesting). Similarly, the MASS database is a new publicly available database and we anticipate future studies will benchmark algorithms against this database.

Ideally, a sleep spindle detection algorithm should correctly detect all true spindles without indicating the presence of additional (erroneous) spindles (an artifact or other class of event erroneously considered to be spindle). In practice, there is a tradeoff compromising between maximizing the detection of true spindles (true positive rate) and minimizing the false assessment of EEG segments as spindles. Essentially this is the case with the closely related algorithms a_7_ and a_8_ proposed in this study. The algorithm a_8_ can typically correctly detect more spindles than a_7_ at the cost of increasing the number of falsely detected spindles (increased false discovery rate). We note that a_6_ and a_3_ are similarly more prone compared to competing algorithms to decide that spindles have occurred in the EEG signal: this causes their true positive rate to be generally higher at the cost of additional false positives. O'Reilly and Nielsen (2014b) envisage that “*most probably, manual [sleep spindle] scoring will progress toward semi-automation benefitting from further advances in signal processing*” an assertion we find plausible. In that sense, if sleep spindle assessment is performed semi-automatically (prior assessment by an algorithm and subsequent checking by an expert) it is beneficial to correctly detect as many spindles as possible, even at the cost of erroneously recording spindles (i.e., increasing sensitivity at the cost of an increased false positive rate). There is probably no universal solution to this problem, and the sensitivity trade-off might need to be a free parameter of sleep spindle algorithms which could be appropriately adjusted by the operator of the algorithm.

We remark that some of the sleep spindle detection algorithms used in this study require more than a single-EEG channel to detect spindles. For example, a_1_ and a_6_ require the use of an additional EEG channel, and a_1_–a_5_ need to be presented with the hypnogram assessment (moreover the algorithm a_5_ explicitly requires stage 2 assessments). We emphasize again that the proposed algorithms in this study (a_7_ and a_8_) have minimal requirements in terms of the input data in order to detect spindles: a single EEG channel is sufficient. Therefore, we argue that these new algorithms may be more readily deployable on databases which have not been scored by experts prior to sleep spindle estimation (no sleep staging requirement). Nevertheless, future studies could further explore whether the use of additional EEG channels and/or hypnogram might increase the sleep spindle detection accuracy.

A critical aspect for comparing algorithms in this application is the definition of TP, TN, FP, FN. In some studies it is not explicitly clear how authors deemed that the automated sleep spindle detector has matched the assessment of an expert in correctly identifying a sleep spindle. There is no clear consensus in the research literature currently; the last column in Table 4 summarizes some of the different approaches that have been used. We agree with Causa et al. (2010) who criticize other studies that the criteria used for algorithmic assessment are not made explicit, and would encourage other researchers to meticulously report the methodology followed to mark their assessments; ideally this methodology should be standardized to facilitate direct comparisons of algorithmic concepts.

Inspection of the results revealed that different sleep spindle detection algorithms have the potential to detect different spindles under different conditions. This would suggest that exploring some data fusion approaches might have good potential in this application. Data fusion in conceptually related applications (combining the outputs of multiple signal processing algorithms which estimate some property of the signal) has shown great promise (Mitchell, 2012; Tsanas et al., 2014; Zhu et al., 2014). In fact, simple combination approaches of the first six sleep spindle detection algorithms used in this study have been previous explored by Warby et al. (2014) but the authors did not report any significant improvement over the single best algorithm; future studies could further explore some principled data fusion frameworks in this application.

## Conflict of interest statement

The authors declare that the research was conducted in the absence of any commercial or financial relationships that could be construed as a potential conflict of interest.

## Supplementary material

Documented Matlab source code is available from the first author's website, and from www.physionet.org.
